# Perceptual Behavior Depends Differently on Pupil-Linked Arousal and Heartbeat Dynamics-Linked Arousal in Rats Performing Tactile Discrimination Tasks

**DOI:** 10.3389/fnsys.2020.614248

**Published:** 2021-01-11

**Authors:** Yuxiang Liu, Shreya Narasimhan, Brian J. Schriver, Qi Wang

**Affiliations:** Department of Biomedical Engineering, Columbia University, New York, NY, United States

**Keywords:** heart rate, heart rate variability, pupillometry, Bayesian decoder, pupil-linked arousal

## Abstract

Several physiology signals, including heart rate and pupil size, have been widely used as peripheral indices of arousal to evaluate the effects of arousal on brain functions. However, whether behavior depends differently on arousal indexed by these physiological signals remains unclear. We simultaneously recorded electrocardiogram (ECG) and pupil size in head-fixed rats performing tactile discrimination tasks. We found both heartbeat dynamics and pupil size co-varied with behavioral outcomes, indicating behavior was dependent upon arousal indexed by the two physiological signals. To estimate the potential difference between the effects of pupil-linked arousal and heart rate-linked arousal on behavior, we constructed a Bayesian decoder to predict animals' behavior from pupil size and heart rate prior to stimulus presentation. The performance of the decoder was significantly better when using both heart rate and pupil size as inputs than when using either of them alone, suggesting the effects of the two arousal systems on behavior are not completely redundant. Supporting this notion, we found that, on a substantial portion of trials correctly predicted by the heart rate-based decoder, the pupil size-based decoder failed to correctly predict animals' behavior. Taken together, these results suggest that pupil-linked and heart rate-linked arousal systems exert different influences on animals' behavior.

## Introduction

Behavioral state, including arousal and attention, profoundly influences brain functions underlying perception, cognition, and behavior (Castro-Alamancos and Connors, 1996; Cano et al., 2006; Niell and Stryker, 2010; McArthur and Dickman, 2011; McGinley et al., 2015a). For example, augmenting cortical response to thalamic microstimulation was found to be heavily suppressed during exploration state as compared to that during resting state in awake rats (Castro-Alamancos and Connors, 1996). In awake mice, it was shown that sensory responses were enhanced and population activity was less correlated in the high arousal state indicated by desynchronized membrane potentials (Reimer et al., 2014).

Several physiological signals, including heart rate and pupil size have been used to index arousal, a state of physiological activation (Wekselblatt and Niell, 2015). Malmo and Davis (1956) found that heart rate co-varied with the gradients of muscle activity, which is indicative of arousal as it reflected the intensity of organism's motivation to do the task, in a mirror tracing task. Heart rate variability was also found to correlate with fluctuating arousal related to emotion and stress (Mather and Thayer, 2018). Therefore, heartbeat dynamics have been widely utilized as an indicator of arousal. Non-luminance mediated change in pupil size has been shown to co-vary with many cognitive factors (Nassar et al., 2012; Hong et al., 2014; de Gee et al., 2020). Recent work has demonstrated that pupil size is able to track cortical state on a moment-by-moment basis (Reimer et al., 2014, 2016; McGinley et al., 2015a; Vinck et al., 2015), suggesting that pupil size is a peripheral index of an arousal system. Previous experimental results suggested the causal effects of arousal on neural responses and behavior (Nassar et al., 2012; Sara and Bouret, 2012; Murphy et al., 2014; Vinck et al., 2015; de Gee et al., 2017; Schröder et al., 2020). For instance, the stimulation of the locus coeruleus—norepinephrine (LC-NE) system not only dilated the pupil, but also improved the behavioral performance of rats performing perceptual tasks (Liu et al., 2017; Rodenkirch et al., 2019), indicating that pupil-linked arousal mediated by the LC-NE system modulates behavior.

The level of arousal is presumably regulated by various neural circuits (Steriade, 1996; Berridge, 2008; de Lecea et al., 2012; Sara and Bouret, 2012; Zagha and McCormick, 2014; Lewis et al., 2015; McGinley et al., 2015b). Several lines of evidence suggested that the LC is a part of pupil-linked arousal system (Joshi et al., 2016; Liu et al., 2017; Rodenkirch et al., 2019). In addition, as varying heart rate and heart rate variability result from the collective regulation of sinus node activity by the sympathetic and parasympathetic systems (Gordan et al., 2015), heartbeat dynamics-linked arousal system is likely to involve both the sympathetic and parasympathetic circuits (also see Discussion). Results from a handful of studies in which ECG and pupil size were simultaneously recorded revealed a positive correlation between heart rate and pupil size, suggesting an overlap between the heartbeat dynamics-linked arousal and pupil-linked arousal systems (Kahneman et al., 1969; Wang et al., 2018). However, the extent to which the two arousal systems differently modulate behavior remains unclear. In addition, previous work involving simultaneous recording of pupil size and heartbeat dynamics were performed in humans, precluding further investigations using genetic and/or invasive manipulations to isolate the effect of individual circuit of the arousal systems on brain functions.

In this study, we aim to examine to what extent the perceptual behavior of awake behaving rats depends differently on arousal indexed by heartbeat dynamics and pupil size. To this end, we simultaneously recorded ECG and pupil size in head-fixed rats performing tactile discrimination tasks. We found that baseline heart rate and pupil size were correlated with behavioral outcomes, indicating both pupil-linked arousal and heart rate-linked arousal modulated behavior in the perceptual tasks. Moreover, baseline heart rate and pupil size co-varied on a trial-by-trial basis, suggesting an overlap between arousal networks indexed by the two physiological signals. To assess the potential difference between the effects of pupil-linked and heart rate-linked arousal on behavior, we constructed a Bayesian decoder to predict animals' behavior from baseline pupil size and heart rate. If the effects of two arousal systems on behavior are identical, the trials on which the decoder correctly predicted behavior based on heart rate should be the same as trials on which the decoder correctly predicted behavior based on pupil size. However, our data showed that this is not the case. Although the number of correctly predicted trials for heart rate-based decoder and pupil size-based decoder was about the same, there was a substantial portion of trials on which the decoder only correctly predicted behavior based on one physiological signal. Furthermore, predictors using both pupil size and heart rate had significantly better performance than predictors using either heart rate or pupil size. Taken together, our results provide strong evidence indicating that pupil-linked arousal and heart rate-linked arousal have different effects on behavior.

## Materials and Methods

Four female albino rats (Sprague-Dawley, Charles River Laboratories, Wilmington, MA; ~225–275 g at time of implantation) were used in this study. All experimental procedures were approved by the Columbia University Institutional Animal Care and Use Committee prior to the study and were conducted in compliance with NIH guidelines.

### Surgical Implantation

All animals used in the study were habituated to experimenters for a minimum of 5 days prior to undergoing surgical procedures to implant a wireless ECG implant (CTA-F40, Data Sciences International, St Paul, MN) and a metal head plate. Animals were single housed after implantation in a dedicated housing facility, which maintained a 12 h light and dark cycle.

The implantation of a head plate was previously described in detail (Schriver et al., 2018, 2020). Briefly, in aseptic surgeries, anesthesia was induced with a Ketamine/Xylazine cocktail (80/5 mg/kg, IP), and buprenorphine (Buprenex, 0.03 mg/kg, SC) was administered as an analgesic. The depth of anesthesia was periodically monitored through reflexes to aversive stimuli and a continuous measurement of heart rate and blood oxygenation using a pulse oximeter (Nonin, Plymouth, MN). Ophthalmic ointment was applied to the eyes during the surgery to prevent the cornea from drying. Fur on the scalp and abdominal area was first shaved and any remaining hair was then removed with depilatory cream. Animals were subsequently mounted in a stereotaxic device using non-penetrating ear bars (David Kopf, CA). The body temperature was maintained at 37°C throughout the procedure through a feedback-controlled heating pad (FHC, Bowdoinham, ME). Alcohol and povidone-iodine solutions were alternately used three times to clean surgical sites. After exposing and cleaning the skull, 6–8 burr holes were drilled in the skull and stainless steel screws (0–80 thread, McMaster Carr, Robbinsville, NJ) were inserted to anchor a head plate (Schwarz et al., 2010). The head plates consisted of custom made aluminum plates which allowed for head-fixation using bilateral pneumatic actuators affixed to a custom restraint box. The center of the head plate was stereotaxically positioned approximately 1 mm posterior to the Lambda and parallel to the skull after which dental cement was applied, anchoring the implant to the bone screws. The scalp wound was then closed with absorbable sutures and treated with antibiotic ointment.

The animal was then dismounted from the stereotaxic frame and placed on a sterile drape covering the heating pad. An incision of ~3 cm was made on the left abdomen. Two additional small incisions were made at the upper right chest and lower left leg to expose the muscle tissue. The transmitter was inserted into the peritoneum and the ECG leads were extended to the right upper chest area and the lower left leg area through a subcutaneous tunnel and sutured to muscle tissue with a non-absorbable suture. The wounds were closed with absorbable sutures and treated with antibiotic ointment. Antibiotic solution (Baytril, 5 mg/kg SC) and additional analgesics (Ketoprofen, 5 mg/kg SC) were administered for a minimum of 5 days postoperatively. The animals began water restriction and subsequent training following a minimum of 15 days of recovery from implantation surgery.

### Behavior

The behavioral apparatus and behavioral task were similar to those described in detail in previous studies (Schriver et al., 2018, 2020). The head-fixation behavioral apparatus was contained in a standard sound and light attenuation chamber (Med Associates, St. Albans, VT). During the tasks, the animals entered the restraint box from the back and placed their head plate into a slot in the front. Two pneumatic cylinders on either side of the head were quickly switched on or off through a foot pedal to fix or release the head plate. A 1 mL syringe body was mounted to a flexible beam and placed directly in front of the animal serving both to deliver water rewards and to measure licking responses via a piezoelectric force sensor. Voltage signals from the force sensor were sampled by a DAQ card (PCI-6259, National Instruments, Dallas, TX).

Throughout the task, the interior of the behavioral chamber was illuminated with an infrared LED, and the animal was remotely monitored with an infrared CCD camera (The Imaging Source, Charlotte, NC). Control of the behavioral task and sampling of animals' behavioral responses were performed by custom-programmed software running on a MATLAB xPC target real-time system (MathWorks, Natick, MA). All behavioral data was sampled at 1 kHz and logged for offline analyses.

#### Tactile Stimulus

**Figure 1:**
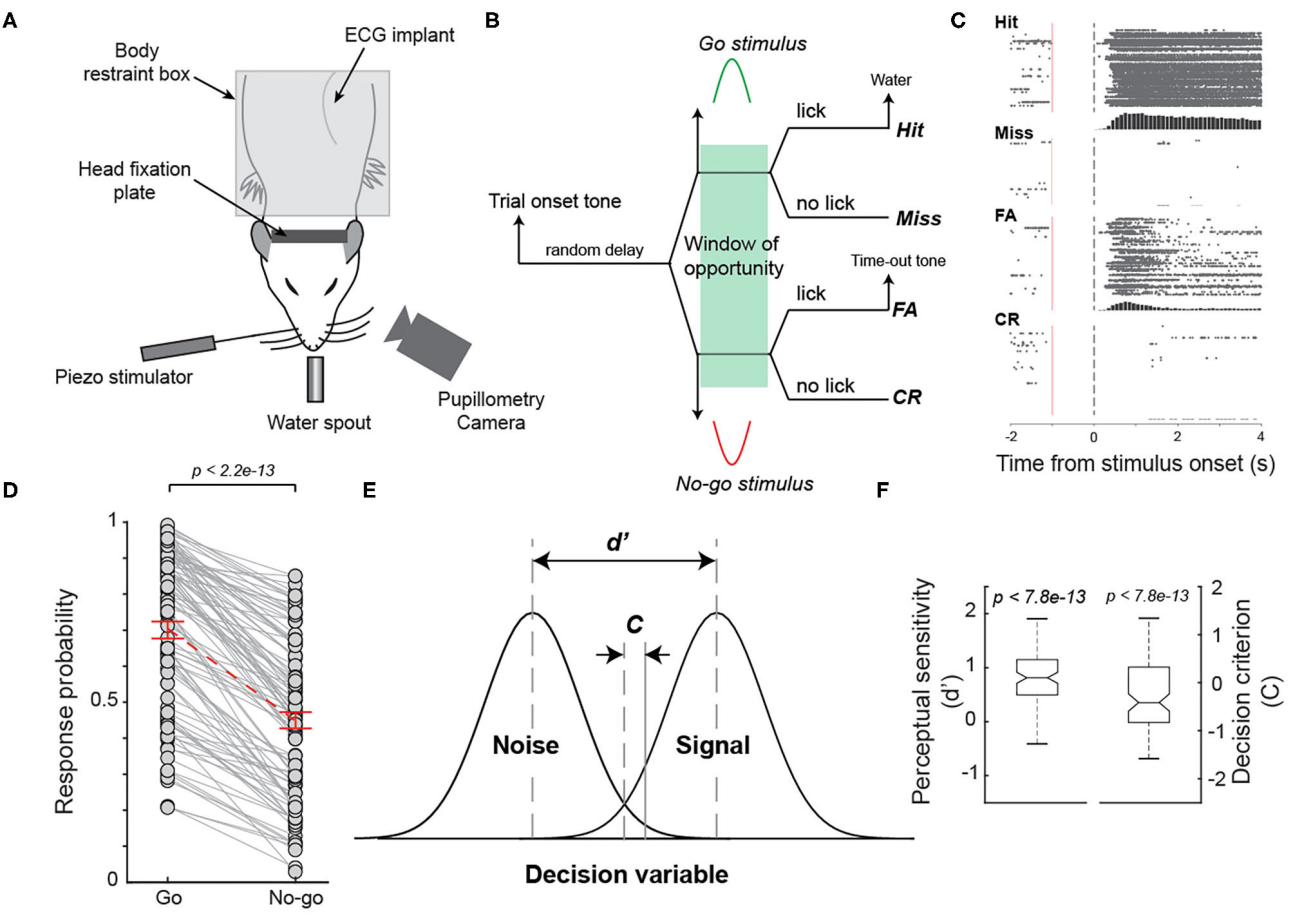
Behavioral performance of rats performing a Go/No-go tactile discrimination task. **(A)** Diagram of experimental set up. **(B)** The diagram of a Go/No-go tactile discrimination task that required animals to discriminate between whisker stimulation in dorsal and ventral directions. **(C)** The raster plot of licks on hit, miss, false alarm, and correct rejection trials of an example session. **(D)** Response probability to the Go stimulus was significantly higher than that to the No-go stimulus across sessions for the animals. *n* = 98 sessions. **(E)** Perceptual sensitivity (d') and decision criterion (c) in the signal detection theory framework. **(F)** Average perceptual sensitivity was significantly >0 while average decision criterion was significantly smaller than 0 across all sessions.

Precise tactile stimuli were delivered using a multilayer piezoelectric bending actuator (PL140, Physik Instrumente, Germany) driven by a custom made high voltage amplifier (Wang et al., 2010; Zheng et al., 2015). To precisely deflect a whisker, a short capillary tube (~15 mm) was bonded to the end of the piezo bending actuator (Figure 1A). The capillary tube was placed ~8 mm away from the right snout, and a whisker of the head-fixed animal was placed inside the capillary tube. The piezo stimulation was oriented such that the whisker could be deflected in the dorsal-ventral direction. To prevent the animal from cueing off the sound of the moving capillary tube during the behavioral task, a second identical piezoelectric bending actuator with capillary tube was placed near the first whisker stimulator without touching any whiskers. This distractor whisker stimulator was programmed to deliver identical stimuli patterns at random time points. To further mask possible auditory cues, a white noise masking sound was delivered through a buzzer placed next to the whisker manipulator.

Whisker deflections with half sinusoidal waveforms with a duration of 50 ms (Figure 1B) in the dorsal direction were randomly designated as Go stimuli, while identical whisker deflections but in the ventral direction were randomly designated as No-Go stimuli. The probability of Go or No-Go stimulus being presented was set to be equal. The peak whisker deflection was ~2 mm, calibrated using a laser micrometer (Metralight, Burlingame, CA).

#### Pupillometry Recording

Recording of the pupil contralateral to the whisker deflection was made using a custom made pupillometry system (Liu et al., 2017), which was triggered at 20 Hz by the xPC target real-time system (MathWorks, MA) that controlled the behavioral task. Pupil images were streamed to a high-speed solid-state drive for offline analysis.

For each video clip, a region of interest (ROI) was first manually assigned. Pupil contour was segmented using the DeepLabCut toolbox (Mathis et al., 2018). Two hundred frames were selected as the training set from pupil videos recorded across different sessions. In each frame, we manually labeled 12 points evenly surrounding the pupil, and set the cropping parameters to improve the training accuracy. The ResNet50 deep network was trained on each frame and utilized to analyze each video clip from all sessions. Circular regression was then applied to fit the automatically labeled points and pupil size was computed as the area within the fitted contour. Approximately 5% of segmented images were randomly selected for visual inspection by experimenters to ensure the accuracy of automatic segmentation. Pupil size during periods of blinks was interpolated using pupil sizes just before and after blinks (Nassar et al., 2012; Schriver et al., 2020). Pupil size was low-pass filtered using a fourth-order non-causal filter with a cutoff frequency of 3.5 Hz (Nassar et al., 2012; Liu et al., 2017).

#### Training and the Go/No-Go Discrimination Task

Water deprivation schedule and procedures of head-fixation habituation were consistent with previous studies (Ollerenshaw et al., 2012, 2014; Bari et al., 2013; Schriver et al., 2018). Briefly, to motivate animals during the tasks, access to water was restricted, i.e., animals did not have access to water in their home cages on training days. However, during the behavioral task, correct responses to a Go stimulus were rewarded with ~60 uL aliquots of water, and supplemental water was provided before returning the animals to the animal facility if water intake during behavioral task was less than the minimal amount needed for physiological functions (Schwarz et al., 2010). During non-training days, animals were given *ad libitum* access to water. The weight of the animals was measured and logged immediately after the task.

The onset of each trial was indicated by a brief “trial onset tone” (300 ms, 6 kHz). Between the trial onset tone and the stimulus presentation, the animal had to wait for a period of random length selected from a 1 to 3.5 s uniform distribution. To discourage the animal from impulsively licking, the last 1 s of the waiting period was a designated “no lick” period during which any premature licks resulted in an additional delay in stimulus presentation selected from a 1–2.5 s uniform distribution (Stuttgen and Schwarz, 2008). Licking within a window of opportunity (0.8 s for 2 animals and 1.3 s for the other two animals; no significant difference between perceptual sensitivities associated with the two windows of opportunity was found) following a Go stimulus resulted in a brief “reward tone” (300 ms, 3 kHz) accompanied by administration of ~60 uL water, while licking within the window of opportunity following a No-Go stimulus triggered a “timeout tone” (5 s, 16.5 kHz) which began a 10 s timeout period. CR and miss behavioral outcomes had no consequences (i.e., not rewarded nor penalized). The time between the reward tone and the onset tone of the next trial was 5.3 ± 0.15 s, while the time between the reward tone and the onset stimulus on the next trials was 8.9 ± 0.14 s. Across all 4 animals, 99 sessions were recorded (24.75 ± 6.4 sessions per animal), and 30,400 trials were performed (307 ± 5 trials per session). One session in which FA rate was higher than hit rate was excluded for further analysis. ECG signals were recorded in all 98 sessions, of which pupillometry was recorded in 69 sessions (17.5 ± 7 sessions per animal).

### Data Analysis

All data analyses were first conducted on individual sessions. Grand averages and standard errors of means were then calculated across sessions for analysis and presentation. For each session the first 20 trials were excluded due to the time required to adjust the pupillometry camera and for experimenters to leave the room.

#### ECG Analysis

To detect R waves to assess heart rate and heart rate variability, raw ECG signals were first high pass filtered (8th-order Elliptic; cutoff frequency = 4 Hz) and low pass filtered (Gaussian window, sigma = 2; cutoff frequency = 250 Hz) (Chavan et al., 2005; Nabian et al., 2017; Kher, 2019). To distinguish R peaks from the ECG signals, the minimum prominence, which measures the threshold level of how much a peak stands out compared to all other adjacent peaks, was computed for each session (Pan and Tompkins, 1985). To determine the optimal threshold value for each session, the total number of distinguished R peaks was assessed recursively as the threshold was incremented gradually by step-size. The optimal threshold was then determined using a downhill simplex algorithm as the threshold at which the descending rate of the R peaks count reached its minimum (Pan and Tompkins, 1985). Once the timing of R peaks was assessed, successive R peak intervals were computed as inter-beats intervals. HR was calculated at time steps of 0.5 s from mean inter-beats interval within a [−0.5 s 0.5 s] time window, whereas HRV was calculated as the standard deviation of inter-beats intervals within the same 1 s window (Shaffer and Ginsberg, 2017).

#### Behavior Analysis

Response probabilities for each session were calculated as the

$$\begin{array}{l} \text{Hit Rate}\\ =\frac{\text{number of hit trials}}{\text{number of trials on which Go stimulus was presented}}\\ \text{FA Rate }\left(\text{FAR}\right)\\ =\frac{\text{number of FA trials}}{\text{number of trials on which No}-\text{Go stimulus was presented}}. \end{array}$$

Perceptual sensitivity (d′) and decision criterion were calculated from hit rate and FA rate as

$$\begin{array}{c} d^{\prime }={\text{Ψ}}^{-1}\left(Hit\;rate\right)-{\text{Ψ}}^{-1}\left(FA\;rate\right)\\ Criterion=-\left({\text{Ψ}}^{-1}\left(Hit\;rate\right)+{\text{Ψ}}^{-1}\left(FA\;rate\right)\right)/2\; \end{array}$$

Where Ψ^−1^ is inverse of the cumulative Gaussian distribution.

Reaction times were computed as the time from stimulus onset to the first lick response within the window of opportunity. Baseline pupil size, HR and HRV were calculated as average pupil size, HR or HRV within 1 s prior to stimulus presentation. To analyze reaction time, perceptual sensitivity, and decision criterion vs. percent of maximum baseline pupil size, HR, and HRV, each session's baseline range was first computed and then evenly broken into 20 bins based on % of maximum baseline computed as

$$\begin{array}{l} \%\text{ of maximum baseline}\\ \;=\;\frac{\text{Baselin}{\text{e}}_{\text{t}}-\text{Baselin}{\text{e}}_{\text{minimum}}}{\text{Baselin}{\text{e}}_{\text{maximum}}-\text{Baselin}{\text{e}}_{\text{minimum}}}\times 100\% \end{array}$$

Each trial was then sorted into one of the bins based on its baseline pupil size, HR, or HRV. Reaction time, perceptual sensitivity, and decision criterion were subsequently calculated for each bin. The loglinear approach was utilized to allow for calculating perceptual sensitivity and decision criterion in bins where hit rate or FA rate equaled 1 or 0, where 0.5 was added to the number of hits and FAs and 1 was added to the number of Go stimulus and No-Go stimulus presentations prior to calculating hit rate and FA rate (Stanislaw and Todorov, 1999; Schriver et al., 2018).

To quantitatively confirm the linear and/or quadratic relationship between HR/HRV and perceptual sensitivity/decision criterion, we performed a regression analysis to evaluate the weights of the linear and quadratic components of each relationship (van den Brink et al., 2016; Schriver et al., 2018). For each session, a polynomial of degree 2 was fitted using least-squares to the relationship between d'/decision criterion vs. HR/HRV baseline. The first- and second-degree beta weights were reported, and statistical tests were used to determine whether these first- and second-degree beta weights significantly differed from zero. To determine the correlation between HR/HRV baseline and pupil size fluctuation, the Pearson's correlation coefficients comparing both HR and HRV baselines to the pupil baselines were calculated and averaged across sessions.

#### Bayesian Inferences

Bayes optimal classifier was utilized to predict behavioral outcome given one predictor, a combination of two predictors, or all three predictors (i.e., pupil size, HR or HRV). The generative models computed probability of animals' response (i.e., responded or withheld response) as

$$\begin{array}{l} \text{ P}\left(\text{Resp}|\text{PA}\right)\;\propto \text{P}\left(\text{PA}|\text{Resp}\right)\;*\text{ P}\left(\text{Resp}\right)\\ \text{P}\left(\text{Resp}|\text{PA},\text{ HR}\right)=\frac{\text{ P}\left(\text{PA}|\text{HR},\text{ Resp}\right)\;*\text{ P}\left(\text{Resp}|\text{HR}\right)}{\text{P}\left(\text{PA}|\text{HR}\right)}\\ \text{ P}\left(\text{Resp}|\text{PA},\text{ HR},\text{HRV}\right)\\ \;=\;\frac{\text{P}\left(\text{PA}|\text{HR},\text{ HRV},\text{Resp}\right)\;*\text{ P}\left(\text{Resp}|\text{HR},\text{HRV}\right)}{\text{P}\left(\text{PA}|\text{HR},\text{HRV}\right)}\; \end{array}$$

where, Resp is behavioral outcome, PA represents baseline pupil size, and HR and HRV represent HR baseline and HRV baseline, respectively. To quantitatively evaluate the conditional probabilities on the right-hand side of the equations, we discretized the baselines and equally divided the baseline range into 15 bins for each session. For a given prior, the conditional probability was calculated as 0 if no trials were sorted into a bin or category. A Laplacian smoothing was then utilized to preserve the complete information in that bin or category (Manning et al., 2008)

$$\begin{array}{l} \hat{\text{P}}\left(\text{A}|\text{B}\right)=\;\frac{{\text{D}}_{\text{B},\text{A}}+1}{{\text{D}}_{\text{B}}+\text{N}} \end{array}$$

Where A and B are two variables, D is the number of observations, and N is the number of possible values of variable B.

We used leave-one-out-cross-validation (LOOCV) to test the performance of the Bayesian decoder in predicting animals' response (Bishop, 2006). More specifically, for each session the likelihood and prior probabilities were computed from all trials except the left-out trial (i.e., test trial). The posterior distribution model was then built from the training set. The maximum a posteriori (MAP) estimation was utilized to output the predicted behavioral outcome for the test trial. After repeating this for every single trial, the percentage of correct predictions over all ground truth responses was calculated for each session. The percent of overlapping trials on which the animal's behavior was correctly predicted by HR and pupil size was calculated as

$$\begin{array}{l} \%\text{ of overlapping trials}=\;\frac{\#\text{ of trials correctly predicted based on both HR and pupil size}}{\left(\#\text{ of trials correctly predicted based on HR}+\#\text{ of trials correctly predicted based on pupil size}\right)/2}\times 100\% \end{array}$$

The normalized overlap was calculated as

$$\begin{array}{l} \text{Normalized overlap}=\;\frac{\text{Percent of overlap}}{\left(\text{Prediction accuracy of pupil based decoder}+\text{prediction accuracy of HR based decoder}\right)/2} \end{array}$$

### Statistics

One-way ANOVA tests were performed to determine whether there exists significant difference among multiple groups. Tukey's honest significance test was performed for all multiple comparisons. One-sample Kolmogorov-Smirnov test was used to verify the normality of the data. For data with a Gaussian distribution, a Student's *t*-test was performed. Otherwise, the Mann-Whitney U-test or the Wilcoxon signed-rank test was used for unpaired or paired samples, respectively.

## Results

To understand the possible correlation between pupil-linked arousal and arousal indexed by electrocardiogram (ECG) signals, we trained 4 animals to perform a Go/No-go tactile discrimination task while simultaneously measuring their pupil size and ECG (Figure 1A). In the tactile discrimination task, the animals were required to respond to a Go stimulus (S+) by licking a lickometer and to withhold a response in the presence of a No-go stimulus (S-) within the window of opportunity (Figure 1B). Either a water reward or a false alarm tone was provided as positive or negative feedback following a lick in response to a Go or No-go stimulus, respectively (Figure 1C). Consistent with previous studies, animals were able to perform the task after 3–6 weeks of training, indicated by greater response probability to a Go stimulus (i.e., hit rate) than response probability to a No-go stimulus (false alarm (FA) rate) (Schriver et al., 2018, 2020) (Figure 1D, hit rate: 0.71 ± 0.023 vs. FA rate: 0.45 ± 0.023, mean ± SEM unless otherwise noted; *p* < 2.2e-13, Wilcoxon signed-rank test, *n* = 98 sessions). To further quantify the behavioral performance, we calculated the perceptual sensitivity d' and decision criterion c, which are measures of ability to discriminate between different stimuli and willingness to respond, respectively, within the signal detection theory framework (Green and Swets, 1966) (Figures 1E,F). The animals achieved an average perceptual sensitivity of 0.84±0.44, which was significantly >0, which is the perceptual sensitivity at the chance level (*p* < 9.0e-18, Wilcoxon signed-rank test, *n* = 98 sessions). Additionally, the average decision criterion was negative (−0.38 ± 0.70; *p* < 6.4e-4, Wilcoxon signed-rank test, *n* = 98 sessions), indicating that the animals were liberal in making decisions. This is likely due to animals being rewarded on hit trials, but not on correct rejection trials (Schriver et al., 2020).

### Heart Rate and Heart Rate Variability Co-varied With Behavioral Performance

**Figure 2:**
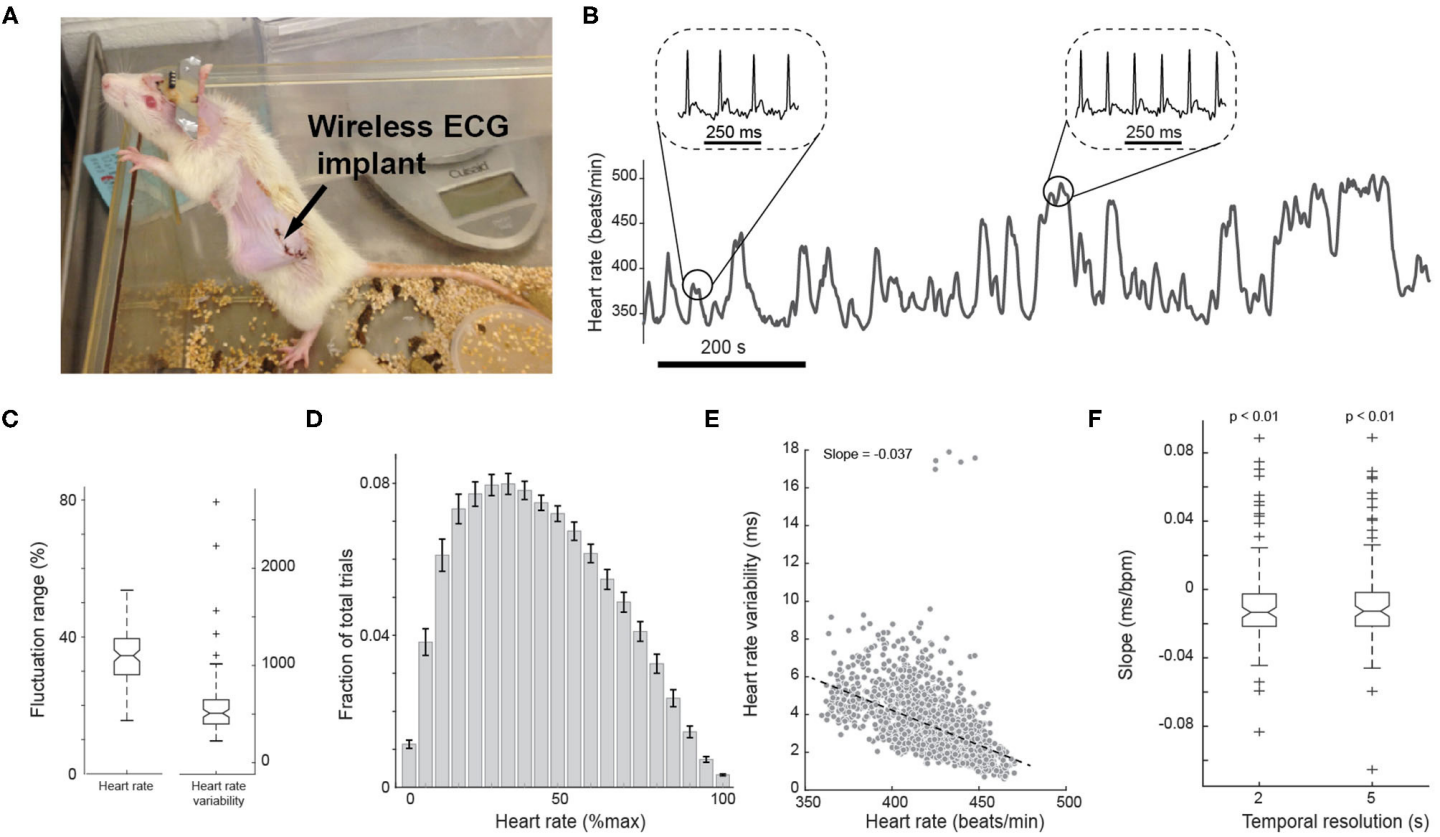
Measurement of ECG from a wireless implant in awake animals. **(A)** Photo of a rat with a wireless electrocardiogram (ECG) implant. **(B)** Representative heart rate fluctuation throughout behavioral tasks. Insets: ECG traces during low and high heart rate periods. **(C)** Boxplots of fluctuation range of heart rate and heart rate variability across all sessions. **(D)** Histogram of heart rate. Error bars indicate SEM. **(E)** Negative correlation between heart rate and heart rate variability of an example session. *n* = 1,148 segments. **(F)** Box plots of the slope of correlation between heart rate and heart rate variability across all sessions.

During behavioral tasks, ECG signals were measured by a wireless ECG implant and were real-time streamed to a telemetry receiver (Figure 2A; see Methods). Heart rate (HR) in awake behaving rats varied dramatically on multiple timescales, swinging between <350 beat per minute (bpm) to >500 bpm (Figure 2B). Heart rate variability (HRV), calculated as the standard deviation of inter-beat-intervals within 1 s periods (see Methods), also fluctuated greatly throughout each session. To quantify these fluctuations, we calculated fluctuation index as the range over the mean value, and found the fluctuation index of HR was 34.3 ± 8.5%. However, HRV exhibited a much larger fluctuation index of 580.6 ± 350.6% as compared to that of HR (Figure 2C). Moreover, the distribution of HR was skewed to its right tail, and the Kolmogorov-Smirnov test (*p* < 0.01) rejected a null hypothesis that the distribution was Gaussian (Figure 2D).

Previous studies have suggested that both HR and heart rate variability (HRV) may be able to index arousal (Azarbarzin et al., 2014; Wang et al., 2018). We first examined the relationship between HR and HRV on a moment-by-moment basis during the task. To this end, we plotted HR vs. HRV for every 2 s throughout each session. We found HR and HRV were negatively correlated on a moment-by-moment basis, resulting in a negative slope of −0.013 ± 0.028 (*p* < 8.5e-3, Wilcoxon signed-rank test, *n* = 98 sessions) (Figures 2E,F). Using a wider time window of 5 s yielded a similar negative correlation (Figure 2F; slope: −0.012 ± 0.028, *p* < 0.01, Wilcoxon signed-rank test; 2 s resolution vs. 5 s resolution: *p* = 0.8, Wilcoxon signed-rank test, *n* = 98 sessions).

**Figure 3:**
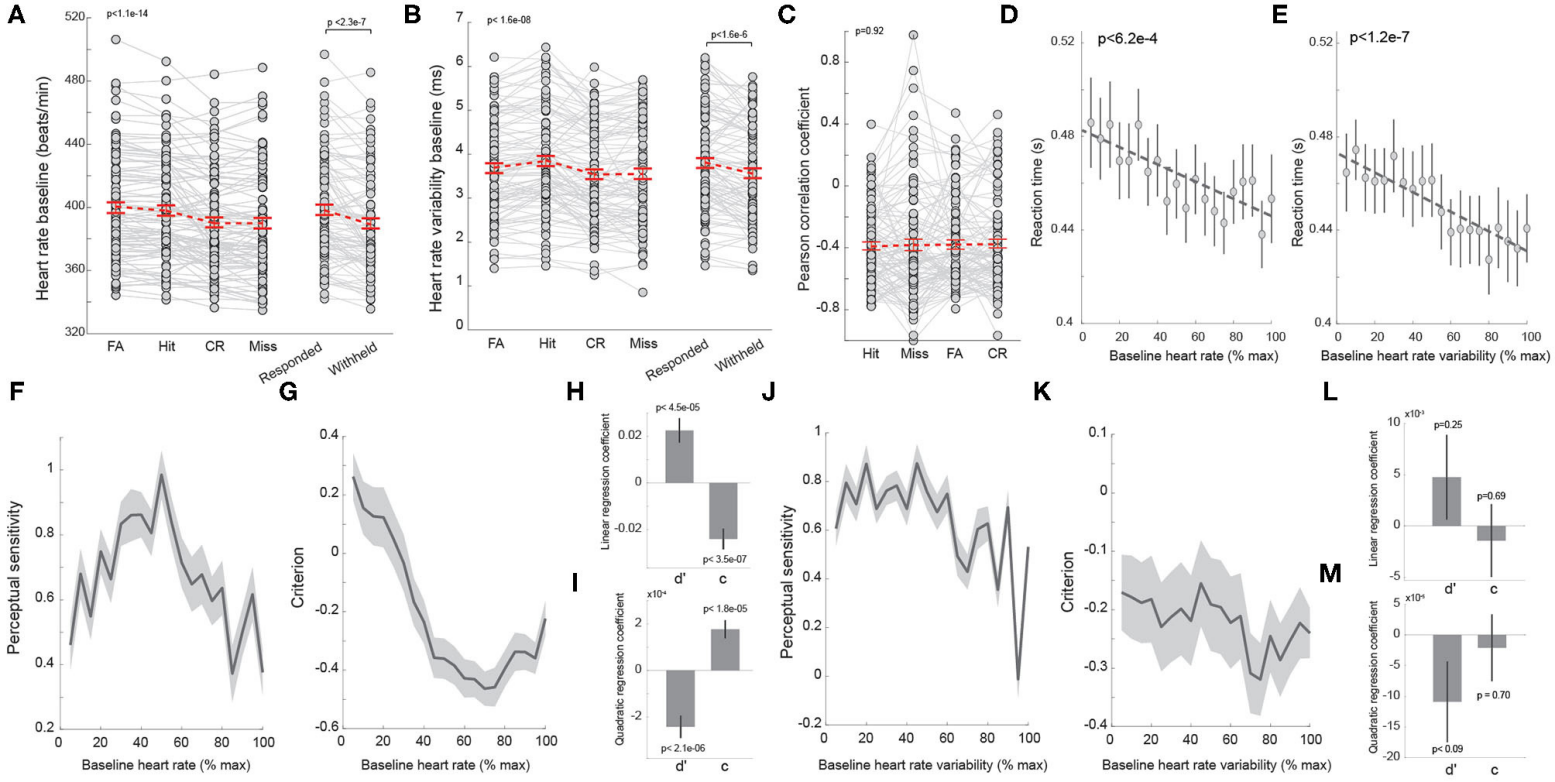
Heart rate co-varies with behavior. **(A)** Average baseline heart rate of trials with different behavioral outcomes. Right: average heart rate of responded and withheld trials. Each dot denotes a session. *n* = 98 sessions. **(B)** Average heart rate variability of trials with different behavioral outcomes. Right: average heart rate variability of responded and withheld trials. Each dot denotes a session. *n* = 98 sessions. **(C)** Pearson correlation coefficient between baseline heart rate and heart rate variability for trials with different behavioral outcomes. *n* = 98 sessions. **(D)** Correlation between baseline heart rate and reaction time. **(E)** Correlation between baseline heart rate variability and reaction time. **(F)** Baseline heart rate exhibited an inverted U-shaped relationship with perceptual sensitivity. **(G)** Baseline heart rate exhibited a U-shaped relationship with decision criterion. **(H,I)** Linear and quadratic regression coefficients of perceptual sensitivity and decision criterion with regard to baseline heart rate. **(J,K)** The relationship between perceptual sensitivity/decision criterion and baseline heart rate variability. **(L,M)** Linear and quadratic regression coefficients of perceptual sensitivity and decision criterion with regard to baseline heart rate variability. Error bars and shaded areas indicate SEM.

To investigate the extent to which HR and HRV co-varied with behavioral performance, we compared baseline HR and HRV prior to stimulus presentation for each behavioral outcome. We found significant differences in baseline HR across different behavioral outcomes [*p* < 1.1e-14, *F*_(3, 94)_ = 25.45, one-way repeated measures ANOVA test, *n* = 98 sessions], with baseline HR being highest for FA trials, followed by hit trials (FA: 399.8 ± 3.4 bpm; hit: 397.9 ± 3.3 bpm) and lowest for miss trials (CR: 390.4 ± 3.2 bpm; miss: 389.9 ± 3.4 bpm; hit vs. miss: *p* < 3e-5, Tukey's HSD *post hoc* test, *n* = 98 sessions). Tukey's *post hoc* test revealed that although baseline HR was higher for FA and hit trials, there was no statistically significant difference between the two behavioral outcomes (FA vs. hit: *p* = 0.3, *n* = 98 sessions) (Figure 3A). Consequently, baseline HR was generally higher on responded trials than withheld trials (responded: 398.6 ± 3.3 bpm vs. withheld: 390.0 ± 3.2 bpm, *p* < 2.3e-7, Wilcoxon signed-rank test, *n* = 98 sessions), consistent with previous findings that pupil-linked arousal is higher on responded trials than withheld trials (Schriver et al., 2018). Taken together, these results suggest that the behavior of the rats depended on HR-linked arousal in the perceptual tasks.

As previous work suggests that HRV may also be an index of arousal, we tested if HRV co-varied with behavioral outcomes in rodents performing our perceptual discrimination behavior paradigm. Similar to HR, we found that HRV prior to stimulus presentation differed significantly across behavioral outcomes [Figure 3B; FA: 3.68 ± 0.11 ms; hit: 3.85 ± 0.12 ms; CR: 3.54 ± 0.11 ms; miss: 3.55 ± 0.12 ms; *p* < 1.6e-8, *F*_(3, 94)_=13.99, one-way repeated measures ANOVA test, *n* = 98 sessions]. In contrast to HR, a *post hoc* test found that HRV was larger on hit trials than FA trials (*p* < 1.2e-6, *n* = 98 sessions). In addition, HRV on both FA and hit trials was larger than HRV on CR and miss trials (hit vs. CR: *p* < 1.1e-7, hit vs. miss: *p* < 2.3e-5, *n* = 98 sessions), resulting in larger HRV on responded trials than on withheld trials (Figure 3B; responded: 3.78 ± 0.11 ms vs. withheld: 3.55 ± 0.11 ms, *p* < 1.6e-6, Wilcoxon signed-rank test, *n* = 98 sessions). However, there was no significant difference in HRV between CR and miss trials (*p* = 0.8, Tukey's HSD *post hoc* test, *n* = 98 sessions). To test if the correlation between HR and HRV was dependent upon behavioral outcomes, we calculated the Pearson's correlation coefficient between HR and HRV for hit, miss, FA, and CR trials. However, we failed to find significant differences in the PCCs across behavioral outcomes (Figure 3C, *p* = 0.9, *F*_(3, 94)_ =0.17, one-way repeated measures ANOVA test, *n* = 98 sessions).

We have previously reported a negative correlation between reaction time and pupil-linked arousal in rats performing tactile discrimination tasks (Schriver et al., 2018). Although the relationship between reaction time and HR has been reported in humans, few studies have examined the relationship in rats performing perceptual tasks. In our animals, reaction time was also found to negatively co-vary with HR (Figure 3D; *p* < 6.2e-4). Interestingly, HRV also exhibit an anti-correlation with reaction time (Figure 3E; *p* < 1.2e-7).

Several studies have demonstrated an inverted U-shaped relationship between pupil-linked arousal and perceptual sensitivity as well as a U-shaped relationship with decision criterion (McGinley et al., 2015a; Schriver et al., 2018). However, whether the relationship between perceptual sensitivity/decision criterion and arousal indexed by HR or HRV exhibits an inverted U-shape or U-shape remains unknown. To directly test this, we binned baseline HR from minimum to maximum for each session and plotted perceptual sensitivity and decision criterion from trials associated with each bin (see Methods). Indeed, the perceptual sensitivity exhibited an inverted U-shaped relationship with baseline HR while decision criterion exhibited a U-shaped relationship with baseline HR (Figures 3F,G). To further quantify these relationships, we regressed perceptual sensitivity and decision criterion against HR and HRV (see Methods). This regression analysis confirmed that perceptual sensitivity and decision criterion had combined linear and quadratic relationships with HR (Figures 3H,I). Interestingly, regression analysis failed to show any statistically significant linear or quadratic relationship between perceptual sensitivity/decision criterion and HRV (Figures 3J–M).

### Heart Rate and Heart Rate Variability Co-varied With Pupil Size

**Figure 4:**
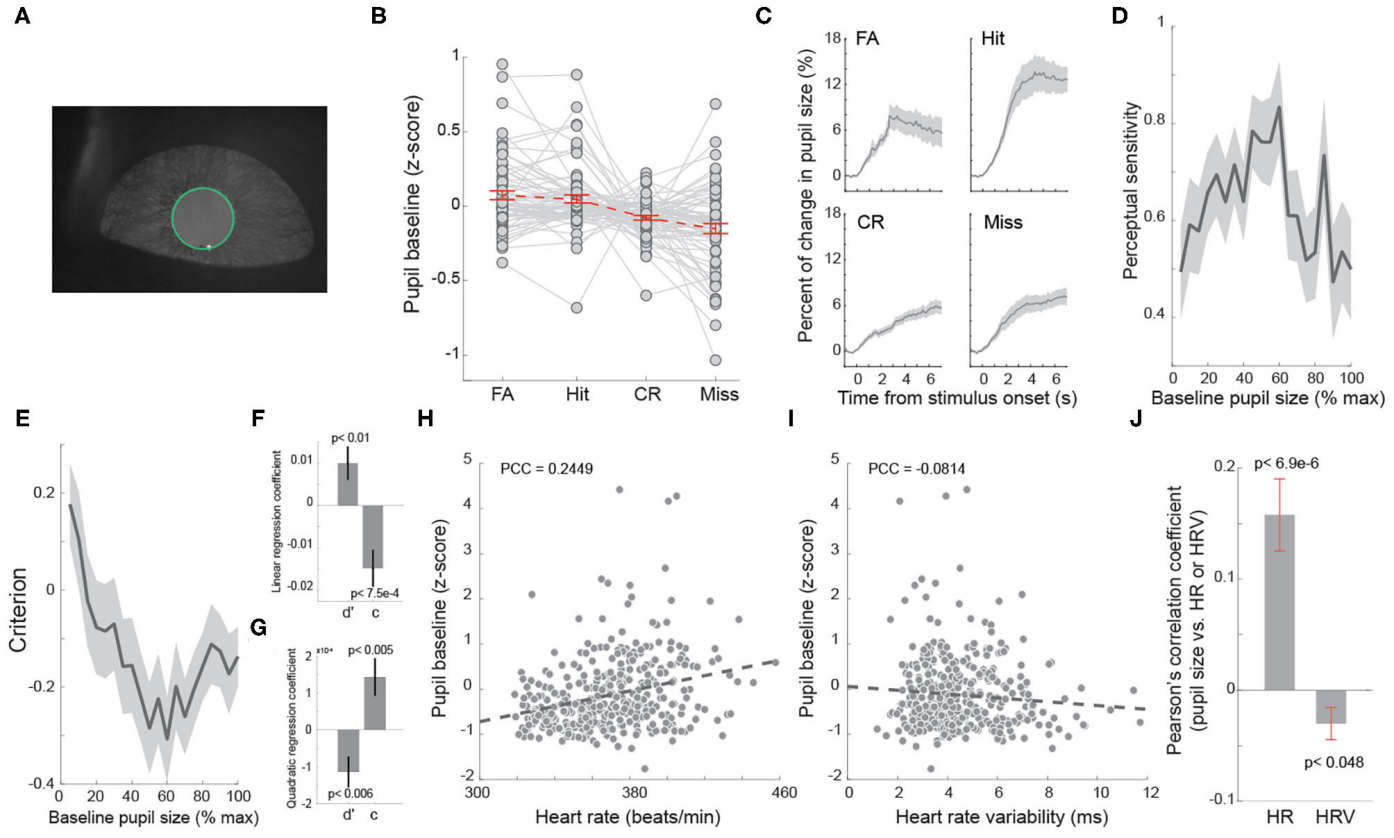
Baseline heart rate co-varied with baseline pupil size. **(A)** Example pupil image. **(B)** Average baseline pupil size of trials with four behavioral outcomes. Each dot indicates a session. *n* = 69 sessions. **(C)** Task evoked pupil dilations associated with four behavioral outcomes. **(D)** Baseline pupil size exhibited an inverted U-shaped relationship with perceptual sensitivity. **(E)** Baseline pupil size exhibited a U-shaped relationship with decision criterion. **(F,G)** Linear and quadratic regression coefficients of perceptual sensitivity and decision criterion with regard to baseline pupil size. **(H)** Correlation between baseline heart rate and pupil size in an example session. *n* = 322 trials. **(I)** Correlation between baseline heart rate variability and pupil size in the example session shown in **(H)**. Each dot indicates a trial in **(H,I)**. *n* = 322 trials. **(J)** Pearson correlation coefficients between baseline heart rate/heart rate variability and pupil size. Error bars and shaded areas indicate SEM.

Our data indicated that behavioral performance was dependent on arousal state indexed by HR. Several previous studies have reported that behavioral performance of rodents performing perceptual tasks depends on pupil-linked arousal (McGinley et al., 2015a; Lee and Margolis, 2016; Schriver et al., 2018). Therefore, it is likely that a correlation between pupil size and HR/HRV exists. We tested if pupil size co-varied with HR and/or HRV in rats performing the tactile discrimination tasks. Consistent with our previous findings (Schriver et al., 2018), there was a significant difference in baseline pupil size across FA, hit, CR and miss trials [Figures 4A,B; *p* < 7.3e-9, *F*_(3, 65)_ = 14.64, one-way repeated measures ANOVA test, *n* = 69 sessions]. Moreover, the animals exhibited unique task evoked pupil dilation for each of the four behavioral outcomes (Figure 4C). As we expected, the perceptual sensitivity exhibited an inverted U-shaped relationship with baseline pupil size while decision criterion exhibited a U-shaped relationship with baseline pupil size (Figures 4D–G). To test if pupil size co-varied with HR or HRV in the behavioral tasks, we calculated the trial-to-trial Pearson's correlation coefficient between baseline pupil size and baseline HR (Figure 4H), as well as baseline HRV (Figure 4I), for each session. We found that there was a significant positive correlation between baseline pupil size and baseline HR (Figure 4J, 0.158 ± 0.032, *p* < 6.9e-6, Wilcoxon signed-rank test, *n* = 69 sessions). In addition, baseline HRV exhibited a weak negative correlation with baseline pupil size (Figure 4J, −0.029 ± 0.014, *p* < 0.047, Wilcoxon signed-rank test, *n* = 69 sessions). Although the magnitude of correlation between baseline pupil size and baseline HRV was smaller than that between pupil size and baseline HR (Figure 4J), further analysis using “*cocor”* package failed to find significance between the two correlations (*p* = 0.35) (Diedenhofen and Musch, 2015).

**Figure 5:**
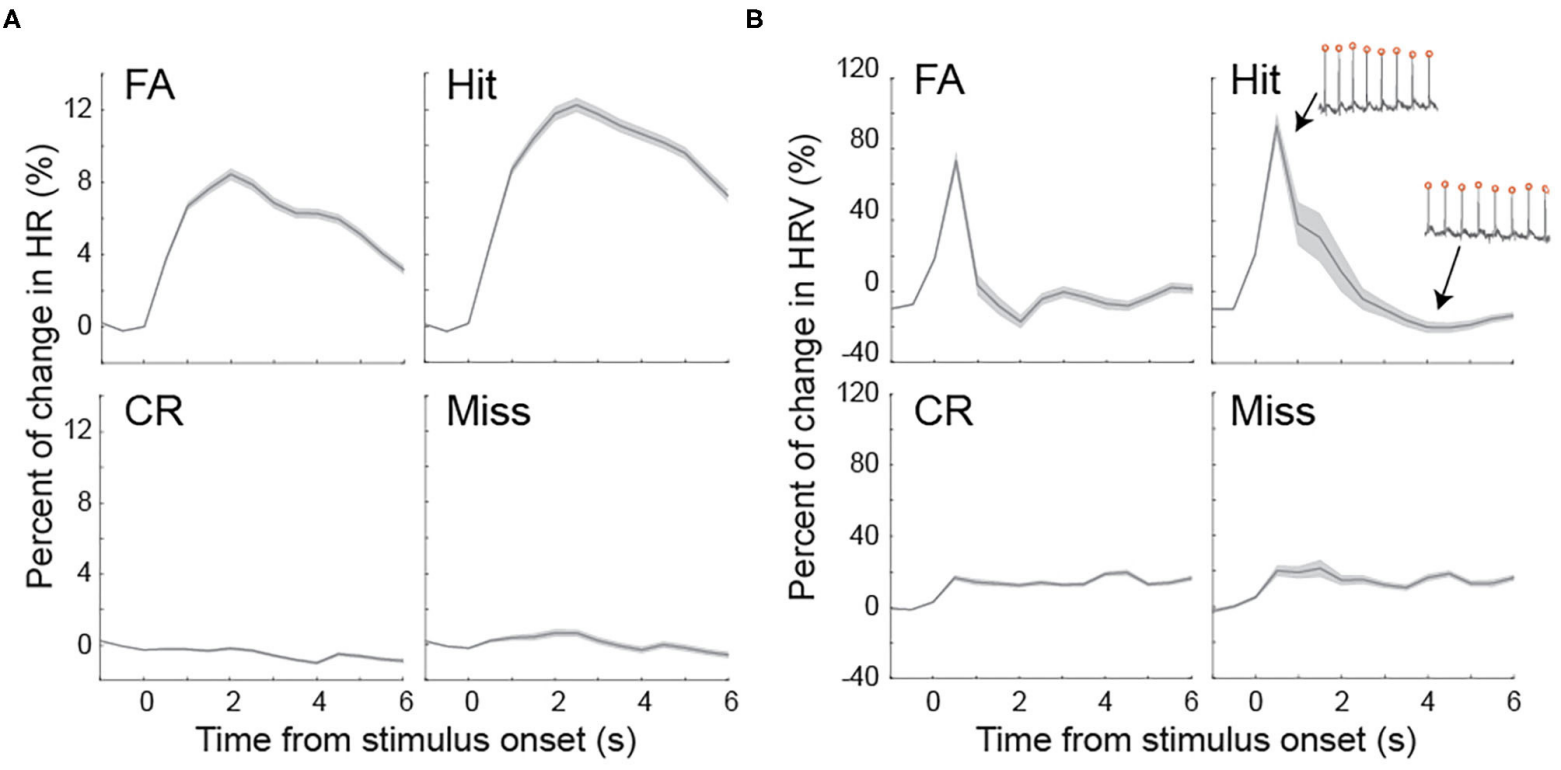
Task evoked fluctuations of heart rate and heart rate variability **(A)** Heart rate dynamics associated with four behavioral outcomes following stimulus presentation. **(B)** Heart rate variability dynamics associated with four behavioral outcomes following stimulus presentation. Inset: example ECG traces with high and low heart rate variability. Shaded areas indicate SEM.

In rats performing perceptual tasks, the pupil rapidly dilated following stimulus onset. Although our data demonstrated a correlation between pupil size and HR/HRV prior to stimulus presentation, the extent to which HR and HRV change following stimulus presentation has not been studied. To test this, we plotted HR and HRV following the onset of stimulus presentation for the four behavioral outcomes. On hit and FA trials, HR rapidly increased and reached peak at approximately 2 s following stimulus onset, whereas HR exhibited relatively small changes on miss and CR trials (Figure 5A). HRV elevated by ~80% from baseline within 1 s following stimulus onset on FA and hit, followed by a sharp decrease (Figure 5B). However, HRV gradually plateaued at approximately 20% on miss and CR trials (Figure 5B).

### Pupil-Linked Arousal and HR-Linked Arousal Differently Modulate Behavior

**Figure 6:**
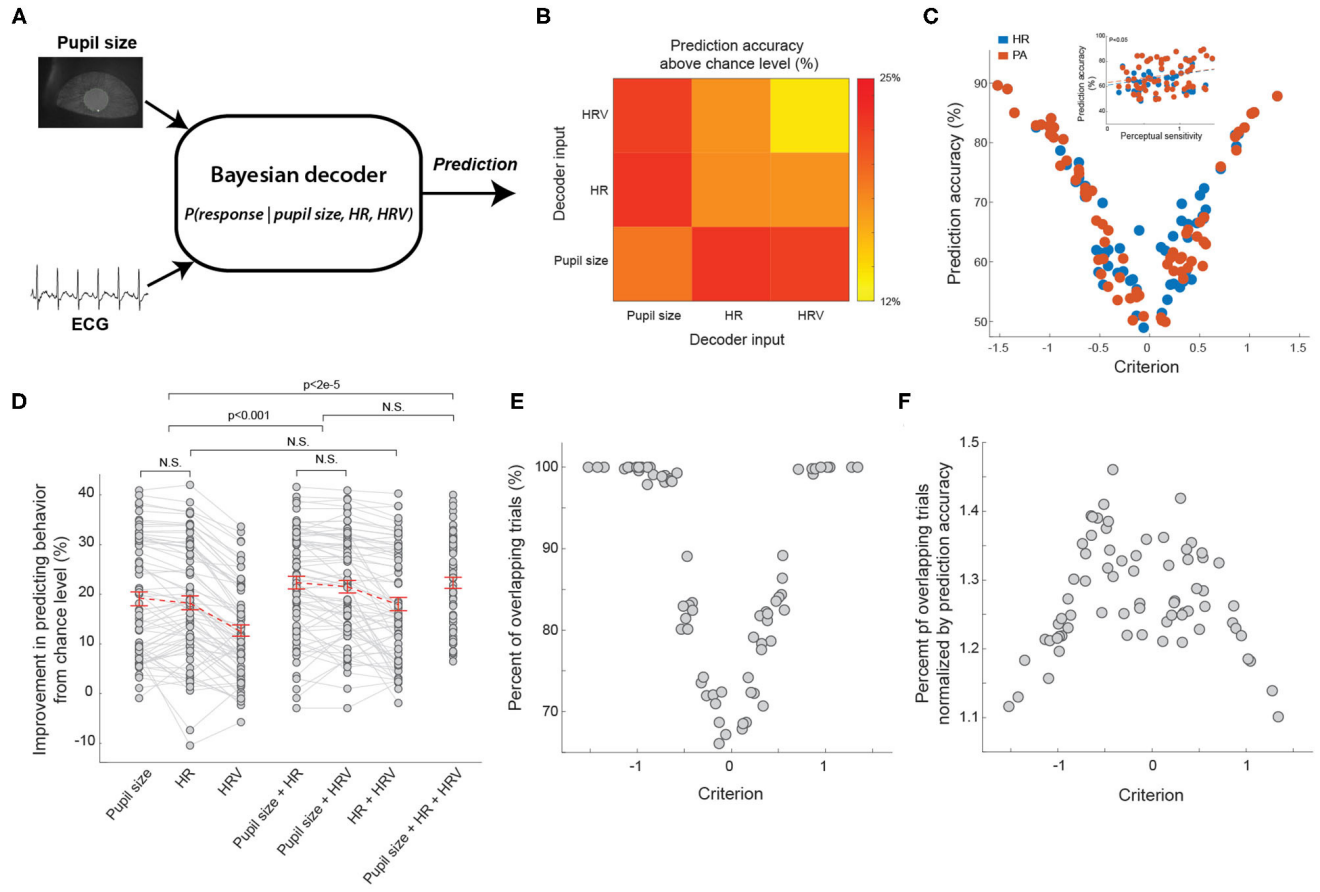
Bayesian decoder to predict animal's behavior from ECG and pupil size. **(A)** The diagram of the Bayesian decoder. **(B)** Average prediction performance based on HR, HRV or pupil size. **(C)** The relationship between the prediction accuracy of Bayesian decoders and decision criterion. Inset: the relationship between the prediction accuracy of Bayesian decoders and perceptual sensitivity. *n* = 69 sessions for each type of decoder. **(D)** Summary of prediction performance based on combination of pupil size, HR, and HRV. *n* = 69 sessions. **(E)** The relationship between percent of overlapping trials and decision criterion. *n* = 69 sessions. **(F)** The relationship between ratio of percent of overlapping trials over prediction accuracy and decision criterion. *n* = 69 sessions. Error bars indicate SEM.

We have showed that both pupil size, HR, and HRV co-varied with behavioral outcomes, suggesting the activation of arousal systems tracked by these physiological signals modulated behavior. However, to what extent these arousal systems overlap remains unclear. It is possible that HR or HRV is a redundant measure of pupil size in indexing arousal state. To investigate this, we constructed a Bayesian decoder to predict animal behavior based on baseline pupil size, HR, HRV, or a combination of these three physiological signals (Figure 6A). If pupil size, HR, and HRV are redundant to each other in tracking arousal state, the performance of the Bayesian decoder in predicting animals' behavior using these physiological signals should be roughly the same. On the contrary, if the activation of arousal systems indexed by the physiological signals differently modulate behavior, the decoder should better predict animals' behavior using a combination of physiological signals indexing different arousal systems. Consistent with previous work, we found the decoder had an above-chance-level accuracy of 19.1 ± 1.4% in predicting whether the animal would respond when using baseline pupil size as an input (Schriver et al., 2018) (Figure 6B). When using HR as an input, the decoder had an above-chance-level accuracy of 18.2 ± 1.4%. Compared with the performance of the decoder using the other two physiological signals, this performance was not significantly different than using pupil size as an input (*p* = 0.10, Tukey's HSD *post hoc* test, *n* = 69 sessions) but was higher than using HRV as an input (12.7 ± 1.1%; *p* < 5e-14, Tukey's HSD *post hoc* test, *n* = 69 sessions). Consistent with our previous work, the accuracy of the decoder was positively correlated with perceptual sensitivity (Figure 6C inset, *p* < 0.05). Intriguingly, we found that the accuracy of both pupil size-based and heart rate-based decoder had a U-shaped relationship with decision criterion, suggesting that both pupil-linked and heart rate-linked arousal profoundly modulated the animals' decision making in the perceptual tasks. This result is consistent with previous human results in which pupil size was related to global modulation of neural gain that generated time-dependent urgency and in turn adjusted decision criteria in perceptual decision making tasks (Murphy et al., 2016).

Although the accuracy of the pupil size-based decoder was approximately the same as that of the heart rate-based decoder, it doesn't rule out the possibility that arousal indexed by pupil size and heart rate has different effects on behavior. To further test this, we constructed a Bayesian decoder using a combination of two signals out of pupil size, HR, and HRV (see Methods). If the decoder using two inputs was better in predicting animals' behavior than using either of them, it is likely that the two inputs carry distinct information. Indeed, we found that by combining pupil size and HR, the decoder had a significantly better above-chance-level accuracy in predicting behavior than only using any one of the three inputs alone (Figure 6D; pupil size + HR: 22.4 ± 1.3% vs. pupil size: 19.1 ± 1.4%, *p* < 5.8e-5; pupil size + HR: 22.4 ± 1.3% vs. HR: 18.2 ± 1.4%, *p* < 4.8e-6, pupil size + HR: 22.4 ± 1.3% vs. HRV: 12.7 ± 1.1%, *p* < 1.5e-20, Tukey's HSD *post hoc* test, *n* = 69 sessions). In addition, decoders using a combination of pupil size and HRV had a comparable performance with those using pupil size and HR as inputs (pupil size + HR: 22.4 ± 1.3% vs. pupil size + HRV: 21.5 ± 1.3%, *p* = 0.06, Tukey's HSD *post hoc* test, *n* = 69 sessions). Interestingly, our data indicated that decoders using HR and HRV as inputs were not significantly better than decoders only using HR (Figure 6D; HR + HRV: 18.03 ± 1.34% vs. HR: 18.28 ± 1.40%, *p* = 0.64, Tukey's HSD *post hoc* test, *n* = 69 sessions), suggesting that the improved accuracy of the Bayesian decoder was not due to noise reduction resulting from combining two inputs. Supporting this notion, decoders using all three physiological signals of pupil size, HR and HRV had a comparable performance in predicting animals' behavior than decoders using only pupil size and HR (Figure 6D; pupil size + HR + HRV: 22.3 ± 1.1% vs. pupil size + HR: 22.4 ± 1.3%, *p* = 0.92, Tukey's HSD *post hoc* test, *n* = 69 sessions).

To further assess the possibly different effects of pupil-linked and heart rate-linked arousal on behavior, we compared the trials on which the pupil sized-based decoder correctly predicted behavior to the trials on which heart rate-based decoder correctly predicted behavior. As we expected, these trials were not completely overlapped. Indeed, the percent of overlapped trials was also dependent upon decision criterion, exhibiting again a U-shaped relationship (Figure 6E). The overlap was smallest when the decision criterion was around 0, and increased to around 100% when the decision criterion increased to ~1 or decreased to ~-1. However, this U-shaped relationship could be due to the U-shaped relationship between decoding accuracy and decision criterion. For example, when the decoding accuracy of pupil size-based decoder and heart rate-based decoder was 100%, the overlap between trials correctly predicted by the two decoders must also be 100%. To control for this possibility, we normalized the percent of overlap by prediction accuracy (see Methods). Our results showed that the normalized overlap exhibited an inverted U-shape, suggesting the effects of the two arousal systems were not redundant on a substantial portion of trials (Figure 6F). Taken together, these results indicated that pupil size and ECG signals carried distinct information about modulation of behavior by different arousal systems, and thus suggest unique effects of the two arousal systems on behavior.

## Discussion

The novelty of this study is that our results, for the first time, provide direct experimental evidence that perceptual behavior in rats performing tactile discrimination tasks depends differently on arousal indexed by heartbeat dynamics than pupil-linked arousal. Heartbeat dynamics are collectively controlled by the sympathetic and parasympathetic nervous systems, both of which are a part of the autonomic nervous system (Kreibig, 2010; Gordan et al., 2015). Varying activities within the sympathetic and parasympathetic systems interact to effect sinus node activity, resulting in fluctuating intervals between heart beats. The sympathetic nervous system is thought to mediate responses to stressors while the parasympathetic system is responsible for relaxing. The sympathetic system originates in the thoracic and lumbar regions of spinal cord (McCorry, 2007), and is responsible for mediating the so called “fight or flight” response in aroused conditions that require body strength and alertness. Therefore, elevated sympathetic tone during high arousal state would lead to elevated heartbeat rate, which subsequently increases the flow of well-oxygenated blood to the brain and skeletal muscles, through binding of NE to adrenergic receptors of cardiomyocytes (Gordan et al., 2015).

The parasympathetic system originates in the brainstem and sacral region of the spinal cord (McCorry, 2007). In contrast to the sympathetic system, the activity of the parasympathetic system maintains homeostatic heartbeat frequency through release of ACh, which bonds muscarinic receptors of cardiomyocytes (Gordan et al., 2015). The function of the parasympathetic system is related to rest and conserving energy during low arousal periods. Therefore, reduced heart rate and blood pressure are usually observed in periods when the parasympathetic system predominates.

Our results are consistent with this notion. We found that heart rate was the higher during responded trials, on which the animals were presumably in high arousal state and were choosing to perform an action for possible rewards (Vinck et al., 2015). On the contrary, heart rate was lower on withheld trials, on which the animals were presumably in low arousal state, evidenced by their low probability of responding even when Go stimuli were presented. Moreover, heart rate exhibited an inverted U-shaped relationship with behavioral performance similar to the classical Yerkes-Dodson curve (Yerkes and Dodson, 1908). Taking together, these results strongly support previous results that heart rate is a reliable indicator of arousal that exerts heavy influences on behavior (Gellatly and Meyer, 1992; Mathias and Stanford, 2003; Wang et al., 2018).

Previous studies have demonstrated that non-luminance mediated fluctuations of pupil size was able to track rapid changes in cortical state (Reimer et al., 2014, 2016; McGinley et al., 2015a; Vinck et al., 2015), and therefore pupil size has been widely considered as a peripheral index of arousal (Nassar et al., 2012; Murphy et al., 2014; Ebitz and Platt, 2015; Lee and Margolis, 2016; Krishnamurthy et al., 2017; Urai et al., 2017; Schriver et al., 2018, 2020; de Gee et al., 2020). It has long been postulated that the LC is the primary brain structure mediating task evoked pupil dilations (Aston-Jones and Cohen, 2005). The LC-norepinephrine system is a major neuromodulatory system that modulates various aspects of information processing in the brain (Martins and Froemke, 2015; Takeuchi et al., 2016; Clewett et al., 2018; Totah et al., 2018; Vazey et al., 2018; Wagatsuma et al., 2018; Rodenkirch et al., 2019; Zerbi et al., 2019; Kaufman et al., 2020). Recent experimental results showed that single unit LC activity was correlated with pupil size (Joshi et al., 2016). In addition, direct micro-stimulation of the LC evoked intensity-dependent pupil dilations (Liu et al., 2017), providing another direct evidence that supports this hypothesis. In the present study, we found that baseline pupil size correlated with heart rate and, to a less extent, with heart rate variability. This may be partly explained by the fact that the LC controls pupil size through both sympathetic and parasympathetic pathways (Steinhauer et al., 2004; Liu et al., 2017). Pupil size is collectively controlled by dilator and sphincter muscles. The activation of the dilator muscle dilates the pupil while the activation of the sphincter muscle constricts the pupil. The activation of the Edinger-Westphal nucleus (EWN), a parasympathetic nucleus, constricts the pupil through its control of sphincter muscle of the pupil via the ciliary ganglion (Steinhauer et al., 2004). The activation of the LC, which inhibits the EWN via alpha-2 adrenergic receptors, would therefore dilate the pupil. The activation of the LC further dilates the pupil through its excitatory control of the sympathetic Superior cervical ganglion (SCG), which in turn controls the dilator muscle of the pupil (Samuels and Szabadi, 2008; Liu et al., 2017). Surgically removing the SCG, therefore eliminating the sympathetic contribution to LC control of pupil size, significantly diminished pupil dilation in response to LC activation (Liu et al., 2017). Therefore, it is plausible that fluctuating sympathetic and parasympathetic tone co-varies with LC control of pupil size, resulting in correlation between pupil size and heart rate that we observed in our experiments.

We found that heartbeat dynamics and pupil size co-varied with behavior, suggesting that arousal indexed by these physiological signals exerts influences on behavior. To test the extent to which the effects of arousal on behavior indexed by HR and pupil size were redundant, we compared the trials on which behavior was correctly predicted by HR or pupil size. If the effects of the two arousal systems were redundant, there should be a complete overlap between the trails. However, our results indicated that this is not the case (Figure 6). The possible difference between the two arousal systems may account for the observed difference between their effects on behavior. The influence of the central nervous system on the autonomic system mainly comes from the hypothalamus (Valenza et al., 2018). The hypothalamus projects to the spinal cord structures and brain stem to regulate the sympathetic and parasympathetic tones through medial forebrain bundle and the dorsal longitudinal fasciculus. The hypothalamus forms heavy connections with brain structures mediating emotional responses to affective or aversive stimuli, including amygdala and prefrontal cortex (Saper, 2000; Phan et al., 2003; Gouveia et al., 2019). Consequently, heartbeat dynamics has long been utilized as an indicator of stress (Kim et al., 2018). In our experimental design, although we did not explicitly induce stress as a factor, it is still possible that timeout periods, indicated by a timeout tone and resulting from false alarm responses, or water rewards, indicated by a reward tone, produced emotional responses in the water deprived animals. Our data indeed support this possibility as we observed dramatic change in heartbeat dynamics following the timeout tone and reward tone (Figure 5). Therefore, arousal indexed by heartbeat dynamics, possibly resulting from brain regions involved in mediating emotional responses, has different effects on behavior than arousal indexed by pupil size, presumably resulting from LC activation. Supporting this notion, we found that, although our Bayesian decoder had comparable performance in correctly predicting animals' behavior based on pupil size and heart rate, the performance of the decoder had significantly better performance when using combined pupil size and heart rate than using either alone. This suggests that pupil size and heartbeat dynamics carry distinct information about different arousal modulation of behavior.

It is worth noting that the prefrontal cortex also forms heavy reciprocal connections with the LC (Aston-Jones and Cohen, 2005). Our data also suggest that when animals are in high arousal or low arousal state, indicated by highly liberal or conservative behavior, the activity in both pupil-linked and heart rate-linked arousal systems was likely to be more correlated, indicated by high overlap between trials on which animals' behavior can be correctly predicted by pupil size and heart rate (Figure 6E). During intermediate arousal state, the activity of the two arousal systems is probably less correlated, as trials on which animals' behavior can be correctly predicted by pupil size or heart rate were less overlapped. It would be intriguing for future studies to use genetic or pharmacological manipulations to tease apart the effect of the different brain structures of the two arousal systems on behavior. This project, to the best of our knowledge, is the first study in which ECG and pupil size were simultaneously recorded in rodents performing perceptual tasks, and thus pave the way for future studies to tackle this important questions using animal models.

## Data Availability Statement

The data supporting the conclusions of this article will be made available to any qualified researchers by the authors, without undue reservation.

## Ethics Statement

The animal study was reviewed and approved by The Columbia University IACUC.

## Author Contributions

QW designed the project. YL, SN, BS, and QW performed the experiments and analyzed the data. YL and QW wrote the manuscript. All authors commented on the manuscript.

## Conflict of Interest

The authors declare that the research was conducted in the absence of any commercial or financial relationships that could be construed as a potential conflict of interest.
